# 

**Published:** 1872-01

**Authors:** 


### American Medical Association.

---

*Office of Permanent Secretary*, WM. B. ATKINSON, 1400 *Pine Street, corner Broad, Philadelphia.*

The Twenty-third Annual Session will be held in Philadelphia, Pa., May 7, 1872, at 11 A. M. Committees are expected to report: On Cultivation of the Cinchona Tree. On the Anatomy and Diseases of the Retina. On the Comparative Pathology and the effects which Diseases of Inferior Animals have upon the Human System, On the Structure of the White Blood Corpuscles. On Vaccinatian. On Skin Transplantation. On the Nature and Process of the Restoration of Bone. On some Diseases peculiar to Colorado. On Corespondence with State Medical Societies. On National Health Council. On Nomenclature of Diseases. On what, if any, Legislative means are expedient and available, to prevent the spread of Contagious Diseases. On American Medical Necrology. On Medical Education. On Medical Literature. On Prize Essays. On the Climatology and Epidemics of all the States.

Physicians desiring to present papers before the Association should observe the following rule:

"Papers appropriate to the several sections, in order to secure consideration and action, must be sent to the Secretary of the appropriate section at least one month before the meeting which is to act upon them. It shall be the duty of the Secretary to whom such papers are sent, to examine them with care, and, with the advice of the Chairman of his Section, to determine the time and order of their presentation, and give due notice of the same."

Secretaries of all medical organizations are requested to forward lists of their Delegates, as soon as elected, to the Permanent Secretary.

Railroad and Hotel arrangements will be announced at an early date.

W. B. ATKINSON.

:o:

MEETING OF THE NEW YORK STATE MEDICAL SOCIETY.—This Society will hold its Annual Meeting at Albany, commencing Tuesday, Feb'y 6th, 1872. It is expected that a large number of delegates and permanent members will be present.

### Books and Pamphlets Received.

Surgeon General's Circular, No. 3.

Proceedings of the Royal Society, Vol. xix, Nos. 123, 124, 125, 126, 127, 128, 129. London, Eng.: William Wesley,

Proceedings of the State Medical Society of Arkansas.

Anæsthesia, Hospitalism, Hermaphroditism, and a proposal to stamp out small pox and other contagious diseases. By Sir James Y. Simpson, M.D. Edited by Sir W. G. Simpson, B.A. New York: D. Appleton & Co. Buffalo: Breed, Lent & Co.

A Clinical Manual of the Diseases of the Ear. By Lawrence Trumbull M.D., with a colored lithograph plate and over one hundred illustrations on wood. Philadelphia: J. B. Lippincott & Co. Buffalo: Breed, Lent & Co.

Fireside Science. A series of popular and scientific essays upon subjects connected with everyday life. By James R. Nichols, A.M., M. D. New York: Hurd & Houghton, 1872. Buffalo: Breed, Lent & Co.

The Mutual Relations of the Medical Profession, its press, and the community. By Dr. Storer, Jr.( Horatio). Boston: James Campbell. 1872.

Remarks upon the diagnosis of ovarian tumors from fibro-cystic tumors of the uterus. By Charles C. Lee, M.D. New York: D. Appleton & Co., 1872.

Inaugural Address, including a Paper on Infant Asylums. By A. Jacobi, M.D. D. Appleton & Co., 1872.

Report of the Hon. Secretary of the Syme Memorial Fund.

Trismus Nascentium. By James S. Bailey, M. D., Albany.

On Vascular Nævi and their treatment by the actual cautery. By B. F. Dawson, M. D.

The Physicians' Annual, 1872. A complete calender for city and country practitioner. Philadelphia: S. W. Butler, M. D., 1872.

Third Annual Report of the Children's Hospital, Boston, 1870 to 1871.

### Medical and Surgical Journal

**PUBLISHED MONTHLY,**

Containing Original Articles, Reports of Medical Societies and Hospitals. Editorial. Reviews, Correspondence, Army News, etc., etc ; including the usual variety of Medical Periodical Publications. Specimen copies sent on application.

Address, Buffalo Medical & Surgical Journal, Buffalo, N. Y.

TERMS OF SUBSCRIPTION, - - $3.00 PER YEAR, IN ADVANCE

